# Evaluation of PD-1 and interleukin-10-receptor expression by T lymphocytes in malignant and benign pleural effusions

**DOI:** 10.1007/s10238-024-01485-y

**Published:** 2024-09-26

**Authors:** B. Mosleh, B. Hammer, A. El-Gazzar, M. Kramer, S. Ayazseven, D. Bernitzky, S. Geleff, Marco Idzko, D. Gompelmann, M. A. Hoda

**Affiliations:** 1 Department of Thoracic Surgery, Comprehensive Cancer Center Vienna, Medical University of Vienna, Vienna, Austria; 2 Division of Pulmonology, Department of Internal Medicine II, Comprehensive Cancer Center Vienna, Medical University of Vienna, Währinger Gürtel 18-20, 1090 Vienna, Austria; 3 Early Life Origins of Chronic Lung Diseases, Research Center Borstel – Leibniz Lung Center, Member of the German Center for Lung Research (DZL), Borstel, Germany; 4 Department of Pathology, Medical University of Vienna, Vienna, Austria

**Keywords:** Pleural effusion, Programmed cell death protein 1, Interleukin-10 receptor, PD-1, IL-10R

## Abstract

PD-1 (programmed cell death protein-1)/PD-L1 (programmed cell death ligand 1) as well as IL-10 (interleukin-10)/IL-10R (interleukin-10 receptor) interactions play a major role in tumor immune evasion in various malignancies. Several studies investigated the expression of PD-1 on T lymphocytes in pleural effusions (PE) in patients with malignant diseases. However, results in malignant pleural effusions (MPE) compared to benign PE (BPE) are underreported. In this prospective study, 51 patients (median age 66 years, IQR 54–78, 47% male) with PE of malignant or benign origin at the Medical University of Vienna between March 2021 and November 2022 were enrolled and divided into three groups according to the cytological results (group 1: MPE [*n* = 24, 47%]; group 2: BPE in malignant disease [*n* = 22, 43%]; group 3: BPE in benign disease [*n* = 5, 10%]). In the cytological samples, T cells were analyzed for the expression of PD-1 and IL-10R via flow cytometry. In MPE, the proportion of PD-1+ T lymphocytes on CD4+ cells was significantly lower than in BPE (40.1 vs. 56.3 in group 1 vs. 3, *p* = 0.019). Moreover, a significantly lower expression of PD-1+ IL-10R+ CD8+ (9.6 vs. 35.2 in group 1 vs. 2, *p* = 0.016; 9.6 vs. 25.0 in group 1 vs. 3, *p* = 0.032) and a significantly higher expression of PD-1-IL-10R-CD8+ T lymphocytes (43.7 vs. 14.0 in group1 vs. 2, *p* = 0.045; 43.7 vs. 23.3 in group 1 vs. 3, *p* = 0.032) were observed in MPE when compared to BPE. The frequency of T cells expressing PD-1 and IL-10R on CD8+ T cells is significantly lower in MPE compared to BPE regardless of the underlying disease indicating a different microenvironment in PE driven by the presence of tumor cells. Our observation spotlights the possible involvement of PD-1 and IL-10R in MPE.

**Supplementary Information:**

The online version contains supplementary material available at 10.1007/s10238-024-01485-y.

## Introduction

The role of the PD-1 (programmed cell death protein 1)/PD-L1 (programmed death ligand 1) axis in cancer surveillance and suppression has been extensively described in the field of immuno-oncology [1, 2]. Thus, targeting PD-1/PD-L1 interactions by immune checkpoint inhibitors (ICI) has been approved for systemic therapy in several tumor entities with clinical breakthrough during the last decade [3–5], whereby PD-L1 tumor proportion score (TPS) has been widely used to guide the selection of patients for immunotherapy in several cancer types [1, 6]. PD-L1 expression is, however, not a clear predictor for response to ICI therapy, as patients with lower PD-L1 expression can also benefit from ICI [7].

Within the heterogeneous and complex landscape of cancer growth and the immune system, PD-1 and its ligand PD-L1 are key immune checkpoint molecules. PD-1, a member of the immunoglobulin superfamily, is expressed on activated T and B lymphocytes, and myeloid-derived dendritic cells, and its function is best characterized in T cell exhaustion. The transmembrane protein PD-L1 is known for its role in immune suppression in autoimmune disease and viral infections as well as in cancer immunology. PD-L1 expression on the surface of tumor cells and antigen-presenting cells (APC) has been described in various malignancies [3, 8, 9], whereby in tumor tissue, a positive correlation between PD-L1 on tumor cells and PD-1 expression on lymphocytes has been demonstrated [10]. The formation of the PD-1/PD-L1 complex was found to mediate tumor-specific T cell exhaustion and apoptosis suppressing the immune-mediated tumor cell destruction resulting in cancer evasion [2, 11, 12].

In addition to the PD-1/PD-L1 axis, the interaction of interleukin-10 (IL-10), a multifunctional immune-regulatory cytokine with immunosuppressive functions, and the interleukin-10 receptor (IL-10R) on regulatory T cells (Tregs) also play a major role in immune evasion of tumors in various malignancies [10, 13]. Furthermore, an enhanced correlation between IL-10R expression and PD-1 levels has been recognized [14]. PD-1 blockade seems to augment the expression of IL-10 receptors by CD8+ T cells, thereby increasing their sensitivity to the immunosuppressive effects of endogenous IL-10 [14–16]. The role of PD-1 and IL-10 in cytological samples, however, remains poorly reported.

Pleural effusions are often a concomitant symptom of various benign or, in 20% of the cases, malignant diseases. While benign pleural effusions (BPE) can result from various causes, malignant pleural effusion (MPE) is the presentation of advanced malignancies. Malignant pleural effusions contain a broad range of cell types including tumor cells and multiple types of immune cells, especially CD4+ and CD8+ T cells, as well as numerous soluble factors such as interleukins. There is limited literature on PD-1 expression in pleural effusions of different cancer types, however [17–20]. The significance of IL-10-mediated production of pleural effusions is also unclear and remains to be investigated [21, 22]. Whether a higher PD-1 expression or an increased interleukin-10 receptor expression can be found in malignant compared to benign pleural effusions has been poorly studied.

The aim of this study was to evaluate PD-1+ and IL-10R expression on CD4+, CD8+ T cells, and FOXp3 regulatory T lymphocytes in pleural effusions of malignant versus benign origin in order to describe the local microenvironment in pleural effusions that may differ in MPE and BPE and may have an impact on therapeutic decisions.

## Methods

This prospective study was conducted to evaluate immunocytochemistry analyses of PD-1 and IL-10 receptor expression on T lymphocytes in pleural effusions due to a malignant or benign underlying disease. In this prospective study, 51 consecutive patients with pleural effusions due to a malignant or benign underlying disease were enrolled. Inclusion criteria was the sonographically confirmed presence of a significant amount of pleural fluid with indication for thoracentesis, pleural catheter, or surgical intervention. All patients were treated at the Department of Thoracic Surgery and the Department of Pulmonology of the Division for Internal Medicine II at the Medical University of Vienna between March 2021 and November 2022. The study was approved by the Ethics Committee of the Medical University of Vienna (EK 2432-2020) according to the Declaration of Helsinki. Written informed consent was obtained by all patients before participating in this study and before study-specific data collection. All interventions were undertaken as part of routine clinical care.

### Study subjects

In this prospective study, 51 consecutive patients with pleural effusions due to a malignant or benign underlying disease with an indication for drainage of the fluid were enrolled. According to the cytological results of the pleural fluid, patients were divided into three groups and compared to each other: cytologically confirmed malignancy (group 1), benign effusion in patients with malignant disease (group 2), and benign effusion in benign disease (group 3).

### Sampling and processing of pleural effusions

Pleural effusions were obtained by thoracentesis, pleural catheters, or surgery (video-assisted thoracoscopy or thoracotomy) at the Department of Thoracic Surgery and at the Division of Pulmonology, Department of Internal Medicine II, Medical University of Vienna, Austria. The pleural fluid, in terms of the clinical indication, was collected for cytological analysis. All samples were processed for routine pathological analyses at the Department of Pathology as well as the Pulmonary Research Laboratory at the Medical University of Vienna, Austria.

For study purposes, approximately 60 ml of pleural effusion was analyzed within 4 h of extraction. The pleural fluids were centrifuged (4 °C, 500 × g, 10 min) and the supernatant removed for future processing. Erythrocyte lysis was performed when appropriate. Cells were re-suspended in phosphate-buffered saline and counted via hemocytometer. 2 × 10^6^ cells were stained for flow cytometric analysis. Pleural effusion cells were first treated with Fc Block (clone Fc1.3216, BD Pharmingen, CA, USA) following surface marker staining of anti-human CD45-BV510 (clone HI30, BD Horizon, CA, USA), CD25-BV785 (clone BC96, BioLegend, CA, USA), CD3-FITC (clone UCHT1, BD Pharmingen, CA, USA), CD210-PE (IL-10R) (clone 3F9, BioLegend, CA, USA), CD8-ECD (clone SFCI21Thy2D3, Beckman Coulter, CA, USA), CD4-PE-Cy7 (clone SK3, BD Pharmingen, CA, USA) and CD279-APC (PD-1) (clone MIH4, BD Pharmingen, CA, USA). For live/dead cell exclusion, cells were stained with Zombie Violet Fixable Viability Kit (BioLegend, CA, USA) and intracellular staining of Foxp3-APC-A700 (clone 259D/C7, BD Pharmingen, CA, USA) was performed using Foxp3 Staining Buffer Set (Invitrogen – eBioscience, Thermo Fisher Scientific, MA, USA) according to manufacturer protocol. Flow cytometric analysis was conducted using a CytoFLEX V5-B5-R3 Instrument (Beckman Coulter, CA, USA) equipped with 405 nm, 488 nm, and 638 nm lasers operating with CytExpert software v2. FlowJo v10 (LLC Software, SD, USA) was used for data analysis.

### Statistical analysis

Categorical data are presented as counts (n) and percentages (%) and scale variables are presented as median and interquartile ranges (IQR). To compare levels of CD4+ lymphocytes, CD8+ lymphocytes, Tregs, and PD-1+ IL-10R+ T cells, chi-square or t-test was used as appropriate. ANOVA or Tukey's test for post hoc analysis was performed to compare the differences. For nonparametric distribution of data, the Kruskal–Wallis test or the post hoc Mann–Whitney U-tests were used. *P*-values < 0.05 were considered statistically significant. Statistical analysis was performed and graphics were generated using the SPSS 27.0 software system (SPSS Inc.).

## Results

### Patient characteristics

In total, 51 patients (24 male, 47%) were enrolled in this prospective analysis. Median age was 66 years (IQR 54–78). Twenty-eight patients (55%) presented with right-sided, and 23 patients (45%) with left-sided pleural effusion (PE). To drain pleural fluids, thoracentesis (27 patients, 53%), insertion of pleural catheters (17 patients, 33%), and surgery (video-assisted thoracoscopy [VATS] or thoracotomy; 7 patients, 14%) were performed. No peri-interventional complications were observed. Forty-six (90%) patients had pleural effusions in the presence of underlying malignant disease including non-small cell lung cancer, pleural mesothelioma, and ovarian cancer as the most frequent malignancies in 16 (31%), 9 (18%), and 6 (12%) patients, respectively. Of these 46 patients with malignant neoplasms, 24 individuals had cytologically confirmed malignant pleural effusions (MPE) (thoracentesis: *n* = 10, pleural catheter: *n* = 5, VATS talc pleurodesis: *n* = 9); while 22 patients were identified with benign cytology (thoracentesis: *n* = 7, repeated thoracentesis/pleural catheter: *n* = 8, thoracoscopy/thoracotomy: *n* = 7). In these 22 patients with malignant disease but benign cytology, 6 patients had the diagnosis of pleural mesothelioma wherein reliable distinction between benign and malignant mesothelial proliferations is dependent on histology and cytology of the pleural fluids has a low sensitivity. In the remaining 16 cases, pleural carcinosis could be confirmed in 4 patients over the course of their disease. (Detailed information on all patients with benign cytology in malignant disease is presented in Supplementary Table S1.) Five (10%) patients had benign pleural effusions (BPE) caused by underlying benign non-infective disease.

Accordingly, patients were divided into three groups for pairwise analyses: group 1 included patients with cytologically confirmed MPE (*n* = 24, 47%); group 2 included patients with BPE in the presence of malignant disease (*n* = 22); and group 3 included patients with BPE in benign disease (*n* = 5). Detailed patient characteristics are displayed in Table 1.

**Table 1:** Patient characteristics

Demographics	n (%)
Age, y, median	66 (IQR 54–78)
*Sex*	
Male	24 (47)
Female	27 (53)
*Diagnostic methods*	
Pleural puncture	27 (53)
Surgery (thoracoscopy/thoracotomy)	17 (33)
Pleural catheter	7 (14)
*Pleural effusions*	
Group 1: Malignant cytology in malignancy	24 (47)
Non-small cell lung cancer	11
Ovarian cancer	5
Pleural mesothelioma	3
Breast cancer	1
Gastric cancer	1
Pancreatic cancer	1
B-cell lymphoma	1
Parotid cancer	1
Group 2: Benign cytology in malignancy	22 (43)
Pleural mesothelioma	6
Non-small cell lung cancer	5
Renal cell carcinoma	5
Breast cancer	4
Ovarian cancer	1
Fibrosarcoma	1
Group 3: Benign cytology in benign disease	5 (10)
*Site*	
Right	28 (55)
Left	23 (45)

### Assessment of T lymphocytes in pleural effusions

The proportion of T cells in pleural effusions was similar in patients with malignant or benign PE whereby CD4+ T cells were predominant in all patient groups. A significant difference in CD4+ T lymphocytes was found between patients with cytologically confirmed MPE and patients with BPE but malignant underlying disease (*p* = 0.031 group 1 vs. 2). The proportion of Tregs of all lymphocytes was low in all patient groups but differed significantly between patients with MPE (group 1) and patients with BPE independent of their underlying disease (group 2 and 3) (*p* = 0.031 group 1 vs. 2; *p* = 0.008 group 1 vs. 3). Proportions of T cells are presented in Table 2.

**Table 2:** Proportion of T cells in pleural effusions between different groups

	Group 1 (cytologically confirmed malignant pleural effusion)	Group 2 (absence of malignant cells in pleural effusion of patients with malignant disease)	Group 3 (benign pleural effusion in patients with underlying benign disease)	p values across all groups
CD4+ T cells (% of CD45+ CD3+ T lymphocytes)	50.5 ± 21.0	49.9 ± 26.9	54.3 ± 31.4	0.849
CD8+ T cells (% of CD45+ CD3+ T lymphocytes)	28.1 ± 23.6	34.3 ± 24.7	34.2 ± 24.5	0.554
Tregs (% of CD45+ CD3+ T lymphocytes)	7.7 ± 3.1	7.2 ± 3.6	3.3 ± 1.4	0.097
PD-1+ T cells (% of CD4+ T cells)	40.1 ± 29.8	49.7 ± 35.5	56.3 ± 46.3	0.039
PD-1+ T cells (% of CD8+ T cells)	43.2 ± 25.9	50.5 ± 34.1	59.0 ± 40.9	0.330
PD-1+ T cells (% of Tregs)	55.3 ± 46.6	62.6 ± 50.1	72.2 ± 66.8	0.236
PD-1+ IL-10R+ T cells (% of CD4+ T cells)	15.0 ± 10.4	24.0 ± 11.5	34.4 ± 14.6	0.209
PD-1+ IL-10R+ T cells (% of CD8+ T cells)	9.6 ± 4.6	35.2 ± 14.8	25.0 ± 24.7	0.019
PD-1- IL-10R- T cells (% of CD4+ T cells)	42.7 ± 35.7	34.6 ± 12.2	32.3 ± 18.1	0.149
PD-1- IL-10R- T cells (% of CD8+ T cells)	43.7 ± 28.7	14.0 ± 4.8	23.3 ± 7.7	0.047

### *Assessment of PD-1*+* T lymphocytes in pleural effusions*

**Fig. 1:**
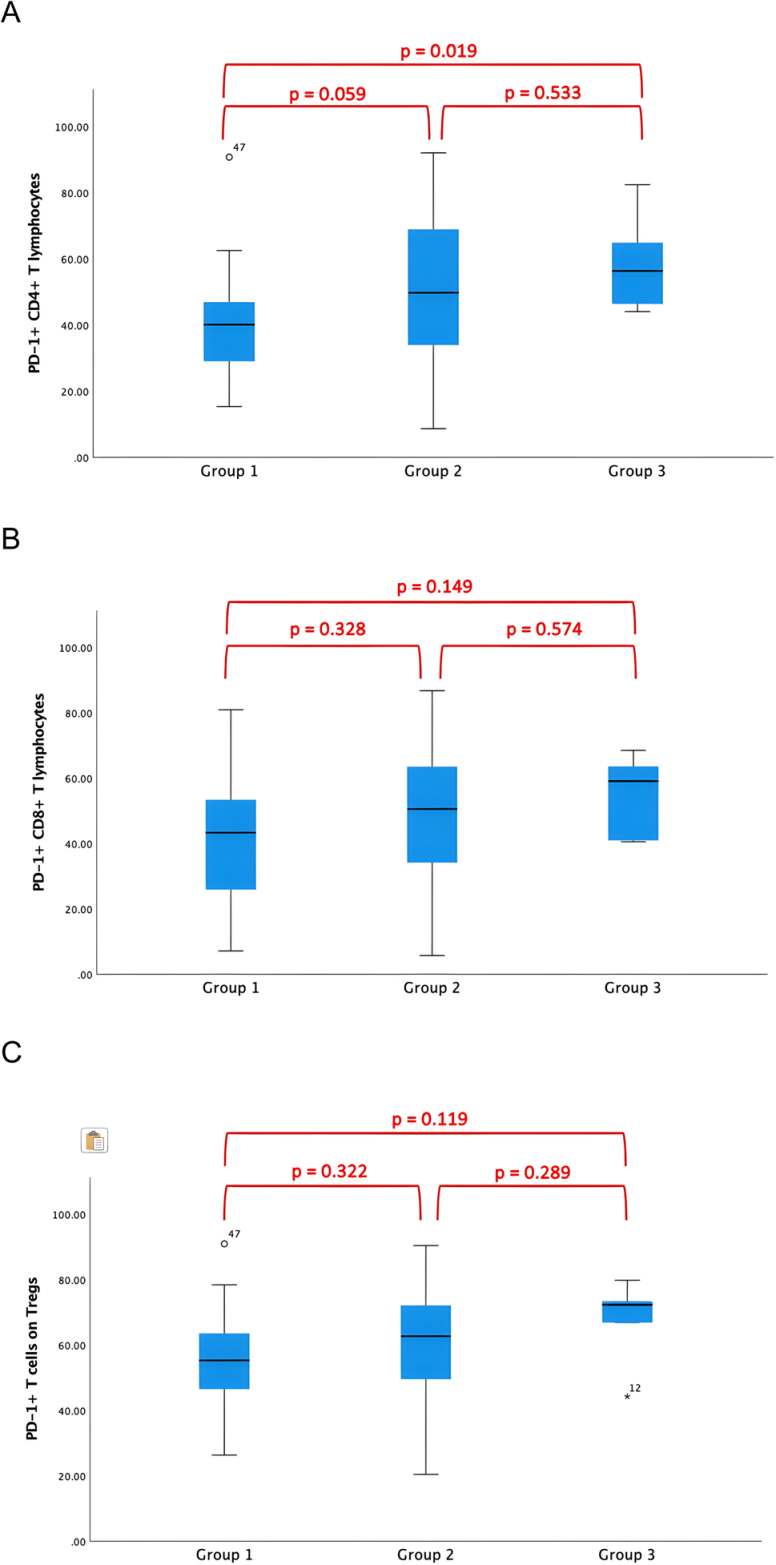
Proportion of PD-1+ T cells on CD4+ T lymphocytes, CD8+ T lymphocytes, and on Tregs was the highest in benign disease and the lowest in in MPE. (**A**) PD-1+ T cells on CD4+ T lymphocytes: *p* = 0.059 group 1 versus 2, *p* = 0.019 group 1 versus 3; (**B**) PD-1+ T cells on CD8+ T lymphocytes: *p* = 0.328 group 1 versus 2, *p* = 0.149 group 1 versus 3; (**C**) PD-1+ T cells on Tregs: *p* = 0.322 group 1 versus 2, *p* = 0.119 group 1 versus 3

The proportion of PD-1 on CD4+ T cells, on CD8+ T cells, and on Tregs was the highest in the PE of patients with benign diseases (group 3) with a median of 56.3% ± 46.3, 59.0% ± 40.9, and 72.2% ± 66.8, respectively, and the lowest in patients with MPE with a median of 40.1% ± 29.8, 43.2% ± 25.9, 55.3% ± 46.6, respectively. In MPE, the proportion of PD-1+ on CD4+ T cells was significantly lower than in BPE (*p* = 0.019 group 1 vs. 3). There were no statistically significant differences in the proportion of PD-1+ on CD8+ T cells and on Tregs. The distribution of PD-1+ on CD4+ T cells, on CD8+ T cells, and on Tregs is shown in Fig. 1. Regarding MPE (group 1), PD-1 expression on T lymphocytes was predominant in effusions due to lung cancer and mesothelioma and was lower in MPE due to ovarian cancer (Table 3).

**Table 3:** Proportion of PD-1 expressing T cells in MPE with regard to the cancer entity

	MPE in patients with lung cancer (*n* = 11)	MPE in patients with ovarian cancer (*n* = 5)	MPE in patients with mesothelioma (*n* = 3)	p values
PD-1+ T cells (% of CD4+ T cells)	43.5 ± 27.3	30.7 ± 27.3	41.7 ± 40.1	0.440
PD-1+ T cells (% of CD8+ T cells)	42.7 ± 25.3	36.4 ± 26.0	56.4 ± 39.9	0.351
PD-1+ T cells (% of Tregs)	56.7 ± 48.6	50.2 ± 48.6	53.8 ± 43.5	0.947

### *Assessment of PD-1*+* IL-10R*+* and PD-1- IL-10R- T lymphocytes in pleural effusions*

**Fig. 2:**
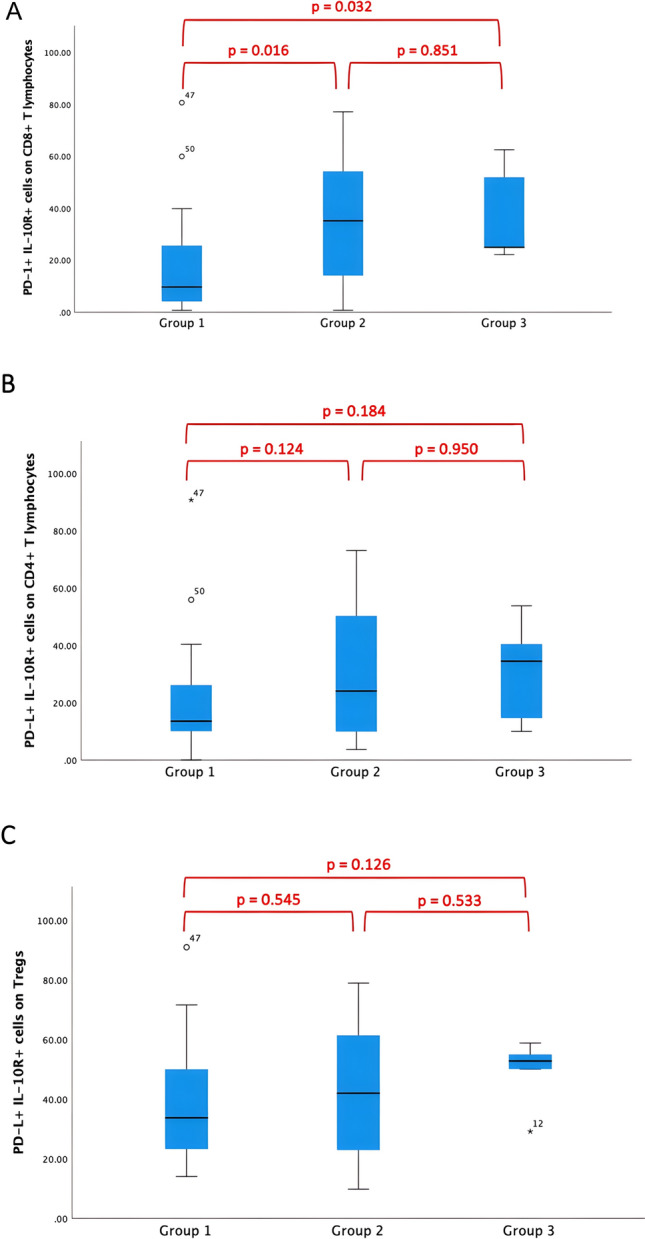
Assessment of PD-1+ IL-10R+ cells on CD4+ T lymphocytes, CD8+ T lymphocytes, and on Tregs in pleural effusions. (**A**) In MPE, the proportion of PD-1+ IL-10R+ cells on CD8+ T lymphocytes was found to be significantly lower than in BPE (*p* = 0.016 group 1 vs. 2; *p* = 0.032 group 1 vs. 3). (**B**) The proportion of PD-1+ IL-10R+ cells on CD4+ T lymphocytes did not show significant differences between groups (*p* = 0.124 group 1 vs. 2; *p* = 0.184 group 1 vs. 3; *p* = 0.950 group 2 vs. 3). (**C**) Similarly, no significant differences could be detected in the proportion of PD-1+ IL-10R+ cells on Tregs (*p* = 0.545 group 1 vs. 2; *p* = 0.126 group 1 vs. 3; *p* = 0.533 group 2 vs. 3)

In patients with MPE, the proportion of PD-1+ IL-10R+ cells on CD8+ T lymphocytes was significantly lower than in patients with BPE independent of their underlying disease (*p* = 0.016 group 1 vs. 2; *p* = 0.032 group 1 vs. 3, Fig. 2A). The proportion of PD-1+ IL-10R+ cells on CD4+ T lymphocytes and on Tregs did not show significant differences between groups. Details are presented in Fig. 2B and C).

**Fig. 3:**
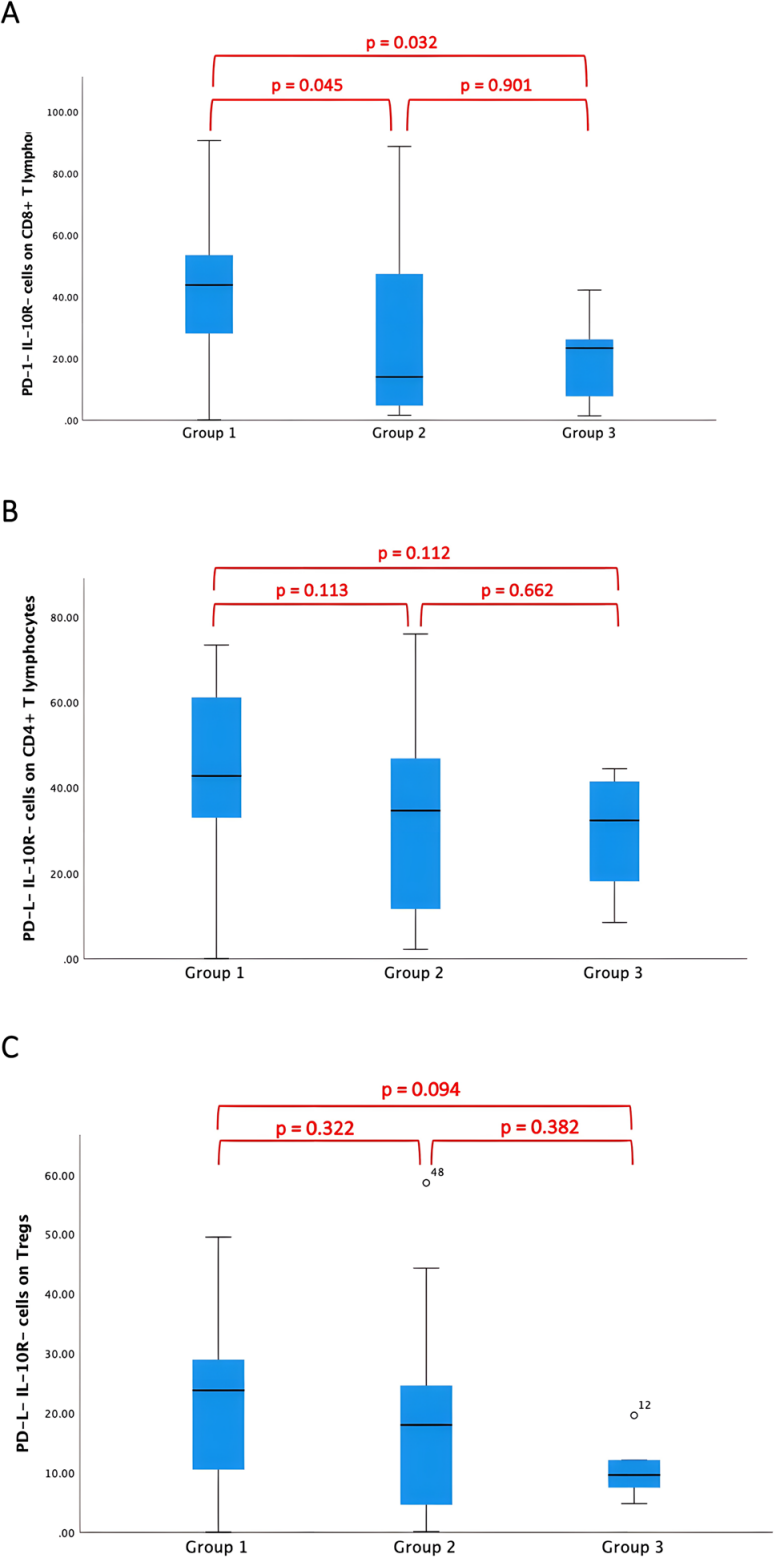
Assessment of PD-1- IL-10R- cells on CD4+ T lymphocytes, CD8+ T lymphocytes, and on Tregs in pleural effusions. (**A**) In MPE, a significantly higher proportion of PD-1- IL-10R- cells on CD8+ T lymphocytes was found when compared to BPE (*p* = 0.045 group 1 vs. 2; *p* = 0.032 group 1 vs. 3). (**B**) There were no significant differences observed in the proportion of PD-1- IL-10R- cells on CD4+ T lymphocytes (*p* = 0.113 group 1 vs. 2; *p* = 0.112 group 1 vs. 3; *p* = 0.662 group 2 vs. 3). (**C**) We also found no significant differences in the proportion of PD-1- IL-10R- cells on Tregs (*p* = 0.322 group 1 vs. 2; *p* = 0.094 group 1 vs. 3; *p* = 0.382 group 2 vs. 3)

Moreover, patients with MPE presented a significantly higher proportion of PD-1- IL-10R- cells on CD8+ T lymphocytes when compared to patients with BPE (*p* = 0.045 group 1 vs. 2; *p* = 0.032 group 1 vs. 3). No significant differences were found between group 2 and group 3 regarding PD-1+ IL-10R+ (*p* = 0.851) and PD-1- IL-10R- cells on CD8+ T lymphocytes (*p* = 0.901), (Fig. 3A).

No significant differences could be detected in the proportion of PD-1- IL-10R- cells on CD4+ T lymphocytes and on Tregs (Fig. 3B and C).

### *Assessment of PD-L1*+* expression on tumor cells*

The median PD-L1 expression on tumor cells (tumor proportion score; TPS) in MPE (*n* = 20) was found to be 5 ± 1% ranging from 0 to 100%.

## Discussion

Malignant pleural effusion (MPE) often occurs in patients with advanced stages of cancer diseases and is associated with dismal prognosis. In MPE, a complex interaction among different immune cells can be observed and several studies have been performed to describe the composition and functional states of infiltrating immune cells in pleural effusions [23]. An increased knowledge about the immunological mechanisms in the microenvironment of malignant pleural effusions may enhance the understanding of potential diagnostic and therapeutic targets.

As immunotherapy provides a therapeutic option for various advanced malignant diseases, several studies focused on the assessment of PD-L1 expression on tumor cells in MPE. It was shown that cytological material is feasible for PD-L1 immunohistochemistry analysis and that the PD-L1 expression correlates between the paired cytological and histological samples [24–26]. It is known that the inefficient antitumoral response is not only due to PD-L1 expression on tumor cells but partly attributed to high levels of PD-1. A few studies confirmed PD-1 expression on T lymphocytes in pleural effusions of patients with lung cancer or mesothelioma [27–29], but there is limited data on PD-1 expression in malignant versus benign pleural fluids. Prado-Garcia et al. reported a significantly higher PD-1 expression on CD4+ and CD8+ T cells in pleural effusions in lung cancer and mesothelioma. This high expression of PD-1 in MPE was comparable to tuberculous PE of chronic infectious disease [19]. Therefore, they hypothesized that PD-1 upregulation on T cells is not induced by the tumor per se but by proinflammatory markers. In our current study, we found the opposite: a significantly higher proportion of PD-1+ CD4+ T lymphocytes in the pleural fluid of patients with benign effusions compared to patients with MPE. Moreover, the PD-1 expression on CD8+ T lymphocytes and Tregs was also found to be the highest in benign effusions but without statistical significance compared to MPE. Of note, in our study, there were no patients with tuberculosis included. In particular, the lack of data on PD-1 expression on immune cells in benign effusions makes it difficult to identify the reasons for PD-1 upregulation in pleural fluids. A similar finding was observed when examining PD-1 expression on T lymphocytes in bronchoalveolar fluids of patients with malignant versus benign disease. The median proportion of PD-1+ CD4+ or PD-1+ CD8+ T cells was found to be higher in patients with interstitial lung disease compared to patients with lung cancer [30]. Although a lower level of PD-1 expression in MPE could be detected when compared to benign effusions, the proportion of PD-1-expressing T lymphocytes was found to be 40–55%. This finding suggests an additional target in therapeutic strategies. Li et al. evaluated the efficacy of intrapleural injection of anti-PD-1 antibodies in MPE in a mouse model and in 9 patients with MPE due to non-small cell lung cancer [31]. After fifteen weeks of intrapleural administration, the control rate of pleural effusion in patients with non-small-cell lung cancer (NSCLC) was 66.7% and the expression of PD-1 on the surface of cytotoxic T lymphocytes in MPE showed a decreasing trend.

The role of IL-10 and its ligand IL-10R in tumor pathogenesis and cancer immunology remains controversial. Tumor-promoting as well as tumor-inhibiting activities are described [32]. A few studies found an enhanced correlation between IL-10R expression and PD-1 levels. Sun and colleagues found that PD-1+ CD8+ T cells upregulate IL-10R expression in patients with malignant melanoma [33].

In this current study, we investigated, for the first time, the co-expression of PD-1 and IL-10R on T cells in pleural effusions. So far, Vahl et al. described a correlation of IL-10R expression and PD-1 levels in the tumor tissue of patients with non-small cell lung cancer and reported a different IL-10R and PD-1 expression between squamous cell carcinoma and adenocarcinoma [10]. In patients with squamous cell carcinoma, PD-1 and IL-10R are co-localized whereas in patients with adenocarcinoma, an accumulation of IL-10R with Foxp3^+^ lymphocytes was found. In this study, we surprisingly found a significantly lower proportion of PD-1+ IL-10R+ cells on CD8+ T lymphocytes in patients with MPE compared to patients with BPE. Interestingly, there was also a significant difference between patients with MPE and those with cancer but cytologically negative pleural effusions. It seems that the presence of tumor cells in the pleural fluid may contribute to low PD-1+ and IL-10R expressions. However, the proportion of PD-1+ IL-10R+ T cells does not support distinguishing between malignant and paramalignant effusions, as in 45% (10/22) of the patients with paramalignant effusions, a pleural tumor involvement was confirmed throughout the course of the disease. However, it should be noted, that the majority of these patients (60%) with malignant disease but benign cytology had the diagnosis of pleural mesothelioma. In all mesothelioma patients, the effusion samples were collected at the time of the diagnostic VATS, wherein the confirmation of malignant cells on tissue specimens could be achieved but the analysis of the pleural effusions showed benign cytology. In pleural mesothelioma diagnosis, accurate deep subpleural tissue biopsies are recommended as effusion cytology for definitive diagnosis has a poor diagnostic yield and remains controversial. As biological differences between the two types of samples in mesothelioma are indicated, the disparity in these cases tends to be more pronounced.

The limitation of this study is the relatively small number of patients with different malignant diseases. Nevertheless, we were able to determine a significantly lower PD-1 and IL-10R expression on T cells in MPE versus BPE.

In conclusion, our study demonstrated a significantly lower frequency of T cells expressing PD-1 and IL-10R in malignant pleural effusions compared to benign effusions and thus a contrary result to the existing literature. As there are several studies evaluating the PD-L1 and PD-1 expression in MPE, only limited data exists on benign pleural effusions. Our findings shed light on the microenvironment, particularly the regulation of PD-1 and IL-10R expression on T cells, in malignant and benign pleural effusions.

## Supplementary Information

Below is the link to the electronic supplementary material.

Supplementary file1 (DOCX 17 KB)

## Data Availability

The datasets generated during and/or analyzed during the current study are available from the corresponding author on reasonable request.
