# Supplementary material for: Evaluation of PD-1 and interleukin-10-receptor expression by T lymphocytes in malignant and benign pleural effusions
Source: Clin Exp Med. 2024 Sep 26;24(1):228. doi: 10.1007/s10238-024-01485-y (PMC11427529; doi:10.1007/s10238-024-01485-y)
Supplement: Supplementary file 1 — Supplementary file1 (DOCX 17 KB) [file 10238_2024_1485_MOESM1_ESM.docx]

**Supplementary Material**

**Supplementary Table S1.** Patients with benign cytology in malignant disease**.** A total, 22 patients were identified with benign cytologic findings in underlying malignant disease (group 2). Of the pleural fluids, 6 were reported as benign in pleural mesothelioma with confirmed malignancy on tissue specimens while in 3 other cases, pleural carcinosis was verified over the course of the disease.

| **Case** | **Diagnosis and the course of disease** | **Detection of pleural malignancy on tissue biopsy** |
| --- | --- | --- |
| 1 | Pleural mesothelioma with no malignant cells on cytology in repeated thoracenteses but confirmed malignant disease on pleural tissue biopsy. | Yes |
| 2 | Pleural mesothelioma with no malignant cells on cytology in repeated thoracenteses but confirmed malignant disease on pleural tissue biopsy. | Yes |
| 3 | Pleural mesothelioma with no malignant cells on cytology in repeated thoracenteses but confirmed malignant disease on pleural tissue biopsy. | Yes |
| 4 | Pleural mesothelioma with no malignant cells on cytology in repeated thoracenteses but confirmed malignant disease on pleural tissue biopsy. | Yes |
| 5 | Pleural mesothelioma with no malignant cells on cytology in repeated thoracenteses but confirmed malignant disease on pleural tissue biopsy. | Yes |
| 6 | Pleural mesothelioma with no malignant cells on cytology in repeated thoracenteses but confirmed malignant disease on pleural tissue biopsy. | Yes |
| 7 | Renal cell carcinoma with no malignancy on cytology in two repeated thoracocenteses but with confirmed pleural carcinosis at VATS tissue biopsy. | Yes |
| 8 | Renal cell carcinoma with no malignancy on cytology in two repeated thoracocenteses but with confirmed pleural carcinosis at VATS tissue biopsy. | Yes |
| 9 | Breast cancer with recurrent pleural effusions and after repeated thoracentesis with radiologically suspected pleural carcinosis but no cytological evidence. | Yes |
| 10 | Fibrosarcoma with radiologically suspected pleural carcinosis and pleural metastases but no cytological evidence for malignancy in the pleural effusion. | Yes |
| 11 | Adenocarcinoma of the lung after anatomic resection with no recurrence of the pleural effusion after thoracentesis. | No |
| 12 | Squamous cell carcinoma of the lung after anatomic resection with no recurrence of the pleural effusion after thoracentesis. | No |
| 13 | Small-cell lung cancer with no evidence of malignancy on cytology in several repeated thoracocenteses. | No |
| 14 | Adenocarcinoma of the lung with no recurrence of pleural effusion after thoracocentesis. | No |
| 15 | Adenocarcinoma of the lung with no recurrence of pleural effusion after thoracocentesis. | No |
| 16 | Breast cancer with post-operative pleural effusion with no recurrence after thoracocentesis. | No |
| 17 | Breast cancer with no evidence of malignancy both on cytology and on VATS pleural biopsy. | No |
| 18 | Breast cancer with no evidence of malignancy on cytology in several repeated thoracocenteses. | No |
| 19 | Ovarian cancer with no recurrence of the pleural effusion after thoracocentesis. | No |
| 20 | Renal cell carcinoma with no evidence of malignancy on cytology in several repeated thoracocenteses. | No |
| 21 | Renal cell carcinoma with no signs of pleural metastasis or carcinosis and with no recurrence of the pleural effusion after thoracocentesis. | No |
| 22 | Renal cell carcinoma with no evidence of malignancy on cytology in several repeated thoracocenteses. | No |
